# Chrysanthemum *CmWRKY53* negatively regulates the resistance of chrysanthemum to the aphid *Macrosiphoniella sanborni*

**DOI:** 10.1038/s41438-020-0334-0

**Published:** 2020-07-01

**Authors:** Wanwan Zhang, Tianwei Gao, Peiling Li, Chang Tian, Aiping Song, Jiafu Jiang, Zhiyong Guan, Weimin Fang, Fadi Chen, Sumei Chen

**Affiliations:** 1 State Key Laboratory of Crop Genetics and Germplasm Enhancement, Key Laboratory of Landscaping, Ministry of Agriculture and Rural Affairs, College of Horticulture, Nanjing Agricultural University, Nanjing, 210095 China; 2 College of Horticulture, Xinyang Agricultural and Forestry University, Xinyang, Henan China

**Keywords:** Biotic, Transcription, Secondary metabolism

## Abstract

Chrysanthemum is frequently attacked by aphids, which greatly hinders the growth and ornamental value of this plant species. *WRKY* transcription factors play an important role in the response to biotic stresses such as pathogen and insect stresses. Here, chrysanthemum *CmWRKY53* was cloned, and its expression was induced by aphid infestation. To verify the role of *CmWRKY53* in resistance to aphids, *CmWRKY53* transgenic chrysanthemum was generated. *CmWRKY53* was found to mediate the susceptibility of chrysanthemum to aphids. The expression levels of secondary metabolite biosynthesis genes, such as peroxidase- and polyphenol oxidase-encoding genes, decreased in *CmWRKY53*-overexpressing (*CmWRKY53*-Oe) plants but dramatically increased in chimeric dominant repressor (*CmWRKY53*-SRDX) plants, suggesting that *CmWRKY53* contributes to the susceptibility of chrysanthemum to aphids, possibly due to its role in the regulation of secondary metabolites.

## Introduction

Aphids are a major group of crop pests that cause severe damage to plants by consuming nutrients from phloem sap^1^. In addition to obtaining nutrients from the phloem, they are also vectors for numerous viruses^2^. Aphids have complex life cycles and produce offspring via cyclical parthenogenesis (CP), making it difficult to control aphids in plants^3^. Plants can employ direct or indirect defenses against herbivore attack. With respect to direct defense responses, plants produce different chemical components, such as phenolics, alkaloids and lignin, to deter herbivores or hinder the growth, development and reproduction of insects^4^. Phenolics, alkaloids and lignin are plant secondary metabolites that confer insect defense to plants^5^. In narrow-leafed lupin, quinolizidine alkaloid biosynthesis is altered in response to aphid predation^6^. Nicotine, an abundant secondary metabolite in tobacco, is a highly toxic chemical to the green peach aphid *Myzus persicae*^7^. Pyrrolizidine alkaloids have been used to deter general insect herbivores, but their relative effects differ between insect species^8^. Lignin is an aromatic heteropolymer synthesized via phenylpropanoid metabolism. Peroxidase (POD) enzymes catalyze the polymerization of monolignols, yielding lignin^9^. Lignin in turn contributes to the defense against aphids^10^. Overexpression of *CmMYB19*, a *MYB* transcription factor, promotes aphid resistance in chrysanthemum by regulating lignin biosynthesis^11^.

WRKY proteins are mainly characterized by one or two highly conserved WRKY domains (WRKYGQK) and a zinc finger motif in the C-terminal region^12^. The domains can specifically bind to W-box sequences ((T/C)TGAC(T/C)) in the promoter regions of target genes to regulate the expression of related genes^13^. Evidence indicates that PAMP-triggered immunity (PTI) and effector-triggered immunity (ETI) defense networks are positively or negatively regulated by *WRKY* transcription factors at the transcriptional level in response to pathogens^14,15^. In *Arabidopsis*, *MPK3/MPK6* phosphorylate *WRKY33* and activate the biosynthesis of phytoalexins in response to *Botrytis cinerea* infection^16^. *AtWRKY33* can also regulate redox homeostasis, salicylic acid (SA) signaling, and ET/JA-mediated defense networks in response to *Botrytis cinerea* infection^17^. In addition, several *WRKY*s are involved in the resistance to herbivores. In *Nicotiana attenuata*, *NaWRKY3* and *NaWRKY6* mediate the resistance to *Manduca sexta* larvae in a JA-dependent manner^18^. Similarly, the tomato genes *SlWRKY72a* and *SlWRKY72b* mediate basal defense against potato aphids^19^. The expression of *WRKY53* and WRKY DNA-binding activities are regulated by SA^20^, which provides a link to the subsequent pathogen response. *WRKY53* plays roles in the biotic stress response and senescence. *Arabidopsis WRKY53* mainly acts as a node in the multilayer regulation of the networks that control senescence and pathogen defense^21^. Furthermore, *AtWRKY53* works in conjunction with *WRKY46* and *WRKY70* by mediating basal resistance against *Pseudomonas syringae* pathogens^22^. However, to our knowledge, there is no information available on the involvement of *WRKY53* in the response to aphids in chrysanthemum.

Chrysanthemum (*Chrysanthemum morifolium* Ramat.) is one of four cut flower species throughout the world and is widely grown for ornamental, tea, and medicinal uses^23–25^. Chrysanthemum plants are frequently attacked by aphids (*Macrosiphoniella sanborni*) during their growth and development^26^, which causes significant economic losses. Transcriptomic changes in response to aphid infestation have been analyzed; where the expression of *CmWRKY53* was significantly upregulated in chrysanthemum fed aphids (unpublished data), inferring that *CmWRKY53* might be associated with the resistance of chrysanthemum to aphids. To test this hypothesis, we cloned the *CmWRKY53* gene in chrysanthemum and studied its function by generating *CmWRKY53* transgenic plants. We showed that *CmWRKY53* mediates the sensitivity of chrysanthemum to aphids by regulating the synthesis of secondary metabolites, highlighting a novel chrysanthemum defense mechanism against aphids.

## Material and methods

### Plant materials and growth conditions

The chrysanthemum cultivar Jinba was obtained from the Chrysanthemum Germplasm Resource Preserving Center, Nanjing Agricultural University, Nanjing, China. Cuttings were transplanted into pots filled with a 1:2 (v/v) mixture of nutrient-enriched soil:vermiculite. The plants were grown in a greenhouse with a relative humidity of 70% and a 16 h/8 h (light/dark) photoperiod. The day/night temperature was 23 °C/18 °C, respectively, and the light intensity was 100 μmol m^−2^ s^−1^. Roots, stems and leaves along with tubular florets and ray florets were sampled for RNA extraction.

### Isolation and sequence analysis of *CmWRKY53*

Total RNA was extracted from leaves using RNAiso reagent (TaKaRa, Tokyo Japan), and reverse transcription was performed using M-MLV reverse transcriptase (TaKaRa) according to the two-step protocol. WRKY53-F/R primers were used to clone the *WRKY53* open reading frame by PCR. The PCR product was purified and cloned into pMD19-T (TaKaRa) for sequencing. Multiple sequence alignments of *CmWRKY53* and its homologs were conducted by DNAMAN 6 software^27^. A phylogenetic tree was then constructed by MEGA 5 software using the neighbor-joining method. The polypeptide sequences of WRKY53 homologs were retrieved from the NCBI website (https://www.ncbi.nlm.nih.gov).

### Expression profile of *CmWRKY53* in response to aphid infestation

Wild-type plants at the 6-8-fully expanded leaf stage were used, and the uppermost leaves and stems of the top three nodes of the shoots were infested with twenty five-instar aphids. Aphid-infested leaves and stems were sampled at different time points after aphid infestation^26^, and non-aphid-infested plants (controls) were sampled at the same time points, with each time point including three individual plants.

### Transactivation assays

The transactivation activity and transcriptional activation domains were analyzed as described previously^28^. The coding region and truncated sequences of *CmWRKY53* were cloned into a pDEST-GBKT7 vector. The resulting pDEST-GBKT7-*CmWRKY53* fusion plasmids were then transformed into cells of yeast strain Y2H Gold (Clontech, Mountain View, CA, USA). Strains introduced with plasmids of pCL1 and/or pDEST-GBKT7 served as positive and negative controls, respectively. The transformants were plated on SD/-Trp media, while the pCL1-harboring strain was grown on SD/-Leu media. Several colonies were then transferred to SD/-His/-Ade media and incubated at 30 °C for three days to determine the activation activity.

### Quantitative real-time PCR (qRT-PCR)

The total RNA of different tissues of aphid-infested samples and control samples was extracted with RNAiso reagent (TaKaRa). cDNA was synthesized using a PrimeScript RT Reagent Kit (TaKaRa). Gene-specific primers were designed using Primer 5 (Supplementary Table S1), and *CmEF1a* was used as a reference gene^29^. The expression of *CmWRKY53* was quantified using SYBR® Premix Ex Taq^TM^ II (Tli RNaseH Plus) (TaKaRa). Three independent biological replicates were used, and the qRT-PCR data were calculated using the 2^−ΔΔCt^ method^30^.

### Subcellular localization of CmWRKY53

The ORF of *CmWRKY53* was cloned into a pMDC43 overexpression vector, generating a construct with the ORF of *CmWRKY53* fused to GFP in the N-terminal region. The plasmids were individually introduced into the *Agrobacterium tumefaciens* strain GV3101, 35S::GFP-CmWRKY53 and 35S::D53-RFP were transiently co-transformed into *Nicotiana benthamiana* leaves, and 35S::D53-RFP acted as a nuclear marker^31^. Expression of GFP and RFP was observed using a Zeiss LSM800 (Germany) laser scanning confocal microscope.

### Generation of *CmWRKY53* transgenic chrysanthemum

The ORF sequence of *CmWRKY53* was first cloned into a pENTR1A gateway vector and then cloned into a pMDC43 overexpression vector, with *CmWRKY53* driven by a 2 × 35S promoter. It has been demonstrated that the ERF-associated amphiphilic repression (EAR) motif functions as a repression domain in plants^32^, and fusion of the EAR repression domain (SRDX) to transcriptional activators is sufficient to convert them into strong repressors^33,34^. Plants expressing a chimeric repressor mimic plants with the corresponding loss-of-function alleles^35,36^. In the present study, CmWRKY53 presented transcriptional activity. To reduce the activities of endogenous and functionally redundant factors, *CmWRKY53* was fused to SRDX to generate a dominant repressor, i.e., *CmWRKY53-SRDX*. The plasmids were introduced into the *A. tumefaciens* EHA105 strain, which was subsequently transformed into chrysanthemum via Agrobacterium-mediated transformation^37^. Overexpression of *CmWRKY53* and the genotype of SRDX transgenic plants were verified by PCR analysis using vector primers and gene-specific primers, and the expression levels of *CmWRKY53* were measured by qRT-PCR using CmWRKY53-RT-F/R primers. All the primers used are listed in Table S1.

### Analysis of aphid resistance in *CmWRKY53* transgenic chrysanthemum

Wild-type and transgenic plants at the 6-8-leaf stage were infested with 5 recently hatched aphid nymphs, and the total number of aphids on the plants was counted at 14 days after infestation. The multiplication rate (MR) and inhibition ratio (IR) parameters were used to quantify the plant resistance. The MR is defined as Ni/5, where Ni represents the total number of aphids on the plants, and the IR is defined 100(NW-NT)/NW, where NW and NT represent the mean numbers of aphids counted at 14 days after infestation on WT and transgenic plants, respectively^37^. Every infestation assay involved 10 plants of each line, and the assay included three biological replicates.

### Transcriptome analysis of *CmWRKY53* transgenic plants

*CmWRKY53*-overexpressing plants (CmWRKY53-Oe2), CmWRKY53-SRDX transgenic plants (CmWRKY53-SRDX2), and wild-type plants at the 6-8-leaf stage were used, the third leaf counted from the apex was collected, and nine individual plants were included in each replicate. The experiment included two biological replicates. Total RNA was extracted as mentioned above. A total of 1.5 μg of RNA of CmWRKY53-Oe2, CmWRKY53-SRDX2 or WT plants was used for RNA-seq (Novogene, China). The library preparation and generation for RNA sequencing followed the methods described by Qi^38^. The DESeq method^39^ was applied to analyze differential gene expression, and the screening threshold was padj <0.05.

### Statistical analysis

SPSS 20.0 software was used to determine statistical significance, and the means and results of the WT and transgenic plants were expressed as the means ± standard errors. The least significant difference (LSD) multiple range test was used to analyze the results after one-way analysis of variance was performed.

## Results

### CmWRKY53 sequence characteristics

**Fig. 1:**
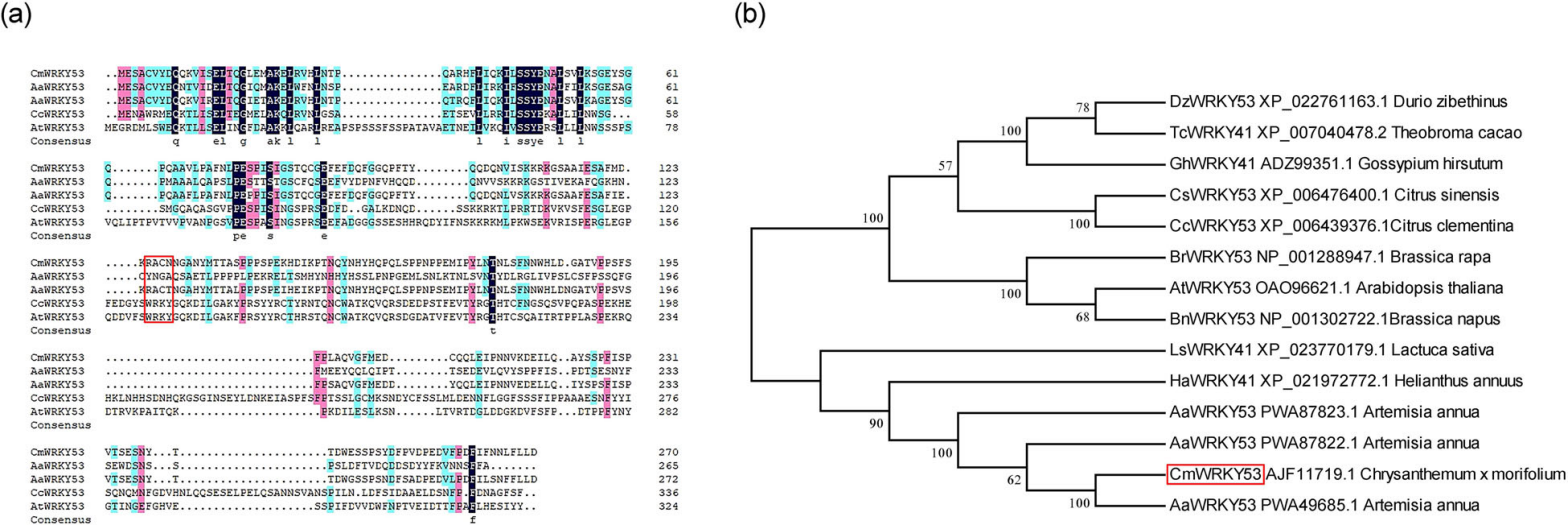
Amino acid sequence of CmWRKY53 and phylogenetic tree of WRKY53s. **a** Amino acid comparison between CmWRKY53 and WRKY53 homologs from other species. **b** Phylogenetic tree of WRKY53s

The sequence of *CmWRKY53* (KM359566), which is 1,114 bp in length and contains an 816 bp open reading frame encoding a polypeptide of 271 amino acid residues, was isolated from Jinba chrysanthemum. Amino acid sequence comparisons showed that the typical WRKY domain is not present in CmWRKY53 (Fig. 1a). Phylogenetic analysis showed that the sequence of CmWRKY53 is most similar to that of *Artemisia apiacea* AaWRKY53 (Fig. 1b).

### Subcellular localization of CmWRKY53

**Fig. 2:**
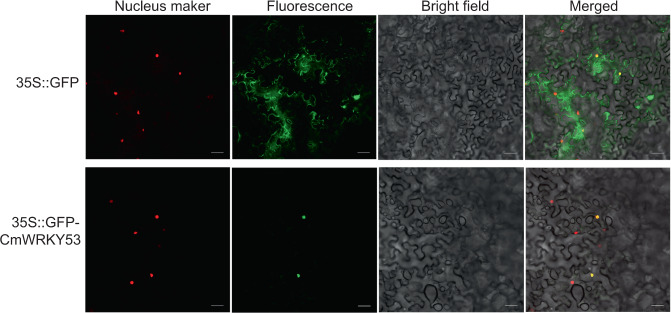
Subcellular localization of CmWRKY53

*A. tumefaciens* transformed with pMDC43-GFP-*CmWRKY53* or a pMDC43-GFP empty vector was infiltrated into the leaves of *N. benthamiana*. GFP fluorescence was detected only in the nucleus of pMDC43-GFP-*CmWRKY53* fusion protein-infiltrated tobacco cells, while GFP fluorescence was evenly distributed throughout the observed tobacco cells that were infiltrated with pMDC43-GFP (Fig. 2). Taken together, these results indicated that CmWRKY53 localized to the nucleus in vivo.

### Transcriptional activity of *CmWRKY53* and analysis of its transcriptional activation domain

**Fig. 3:**
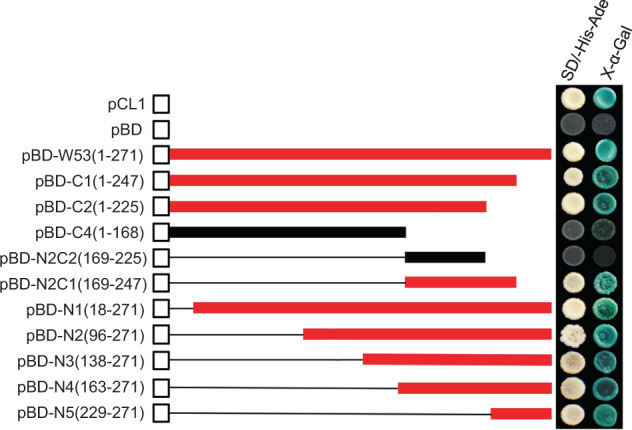
Transactivation assay of CmWRKY53

The pDEST-GBKT7-*CmWRKY53* plasmid was transformed into the yeast strain Y2H to determine the transcriptional activity of CmWRKY53. The Y2H Gold yeast transformed with pDEST-GBKT7-*CmWRKY53* grew normally in double-deficient media, the negative control yeast transformed with pDEST-GBKT7 did not grow, and the positive control yeast transformed with pCL1 grew normally (Fig. 3). The results indicated that the whole CmWRKY53 protein is transcriptionally active. To identify the specific transactivation region of the CmWRKY53 protein, different truncated segments of CmWRKY53 from the C- and N-termini were cloned into pDEST-GBKT7. The results showed that the yeast strains containing the recombinant plasmids of the pDEST-GBKT7-*CmWRKY53* (1-168 aa) and pDEST-GBKT7-*CmWRKY53* (169-225 aa) fragments did not grow on the double-deficient media, but fragments of 1-225 aa and the remaining segments all grew normally (Fig. 3). Together, these results suggest that the activation domain is located within the 229-271 aa region at the C-terminus and that the intact protein of approximately 168 aa is important for activation activity.

### Expression profiles of *CmWRKY53* in different tissues in response to aphid infestation

**Fig. 4:**
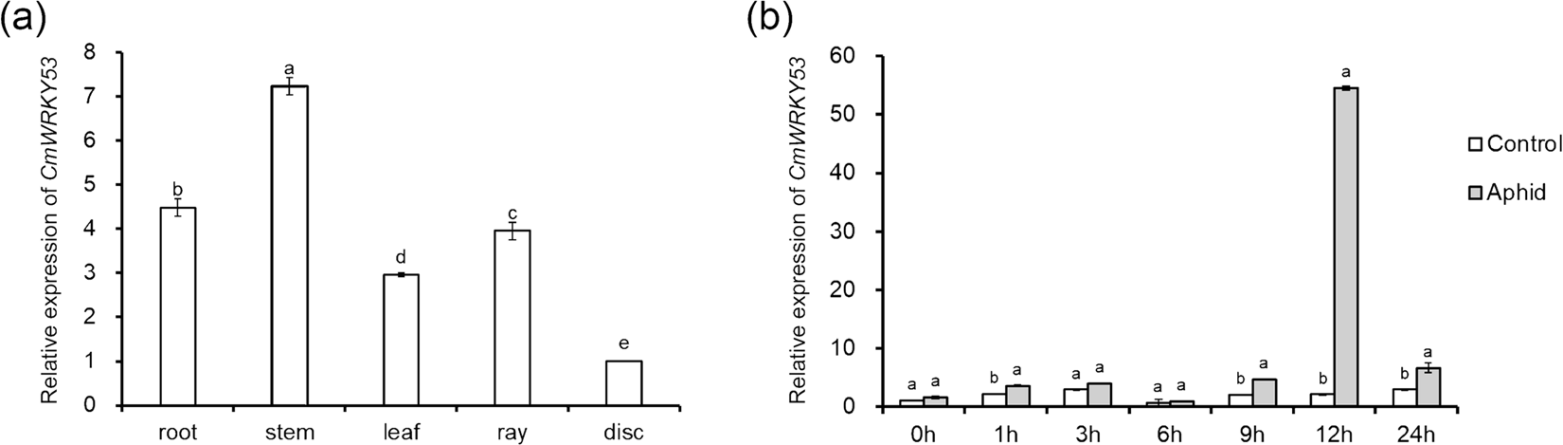
Relative expression level of CmWRKY53. **a** Relative expression level of *CmWRKY53* in different tissues of Jinba. **b** Transcriptional profiling of *CmWRKY53* in response to aphid infestation

The relative expression levels of *CmWRKY53* were monitored in the root, stem, leaf and flower tissues of chrysanthemum. The results demonstrated that the relative expression levels were highest in the stems, followed by those in roots and leaves, while the disk florets presented the lowest levels of expression (Fig. 4a). The relative expression of *CmWRKY53* increased by 1.28-fold at 9 h after aphid infestation and by 25.11-fold at 12 h after aphid infestation compared with that in the non-infested plants (Fig. 4b).

### CmWRKY53 contributes to the aphids susceptibility of chrysanthemum

**Fig. 5:**
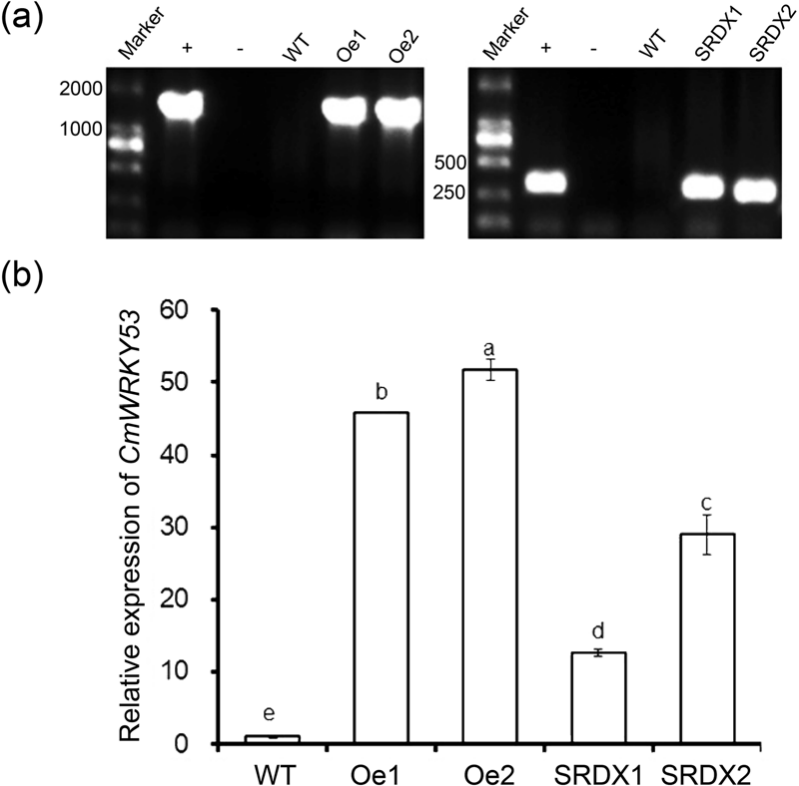
Identification of CmWRKY53 transgenic plants. **a** PCR-based identification of pMDC43-*CmWRKY53* and pSRDX*-CmWRKY53* transgenic lines using vector- and gene-specific primers. For the positive control, the pMDC43-*CmWRKY53* and pSRDX*-CmWRKY53* plasmid were used as template, and for the negative control, no template was added. **b** Relative expression levels of *CmWRKY53* in the transgenic plants

**Fig. 6:**
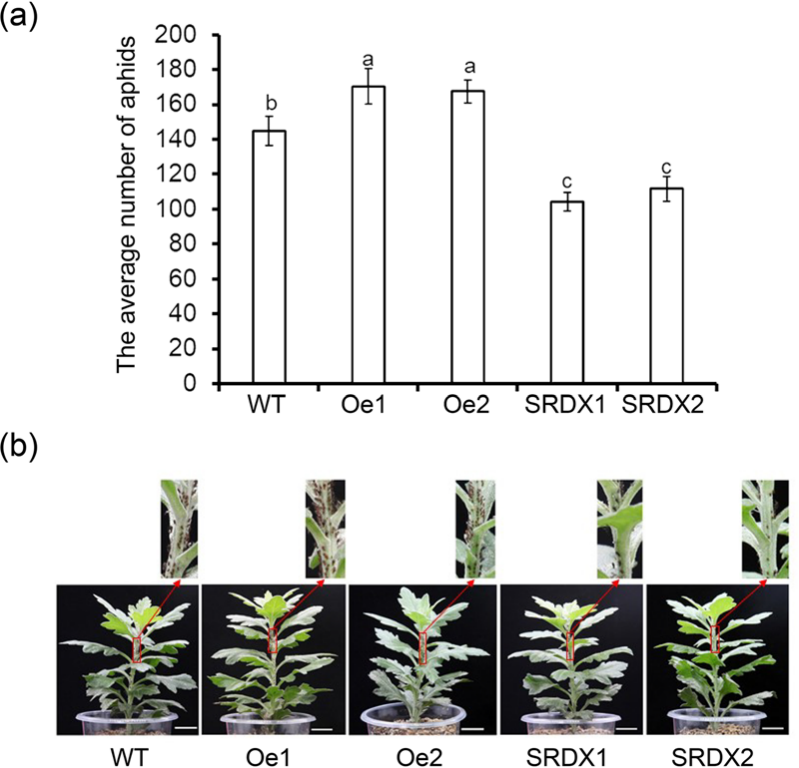
Proliferation of aphids on WT and transgenic lines at 14 days after inoculation, bar = 2 cm. **a** The average number of aphids on WT and transgenic lines. **b** Image of aphids on WT and transgenic lines

To determine the function of *CmWRKY53*, overexpression and gene-silenced transgenic chrysanthemum lines were obtained. Transgenic overexpression lines were verified by PCR amplification using a 35S forward primer and a reverse *CmWRKY53* gene-specific primer, and *CmWRKY53-*SRDX lines were verified using a forward gene primer and an SRDX reverse primer. The expected bands were present for the transgenic lines and the positive control samples but not for WT or negative control samples (Fig. 5a). The transgenic plants were further verified using qRT-PCR (Fig. 5b). Aphid infestation assays showed that the number of aphids on the WT plants was lower than that on *CmWRKY53*-overexpressing plants but higher than that on CmWRKY53-SRDX gene-silenced transgenic plants (Fig. 6). The aphid MR on the WT plants was 28.92, while on the *CmWRKY53*-overexpressing lines, the rates were 34.06 and 33.48, and those in *CmWRKY53*-SRDX gene-silenced lines were 20.86 and 22.34. The IRs for *CmWRKY53*-overexpressing lines were −17.77% and −15.77%, whereas they were 27.87% and 22.75% for the SRDX lines (Table 1), suggesting that *CmWRKY53* contributed to the sensitivity of chrysanthemum to aphids.

**Table 1:** MR and IR percent of aphids in WT and *CmWRKY53* transgenic lines 14 days after the infestation

	WT	Oe1	Oe2	SRDX1	SRDX2
MR	28.92	34.06	33.48	20.86	22.34
IR(%)	0.00	−17.77	−15.77	27.87	22.75

### Expression profiles of genes involved in secondary metabolism in *CmWRKY53* transgenic plants

**Fig. 7:**
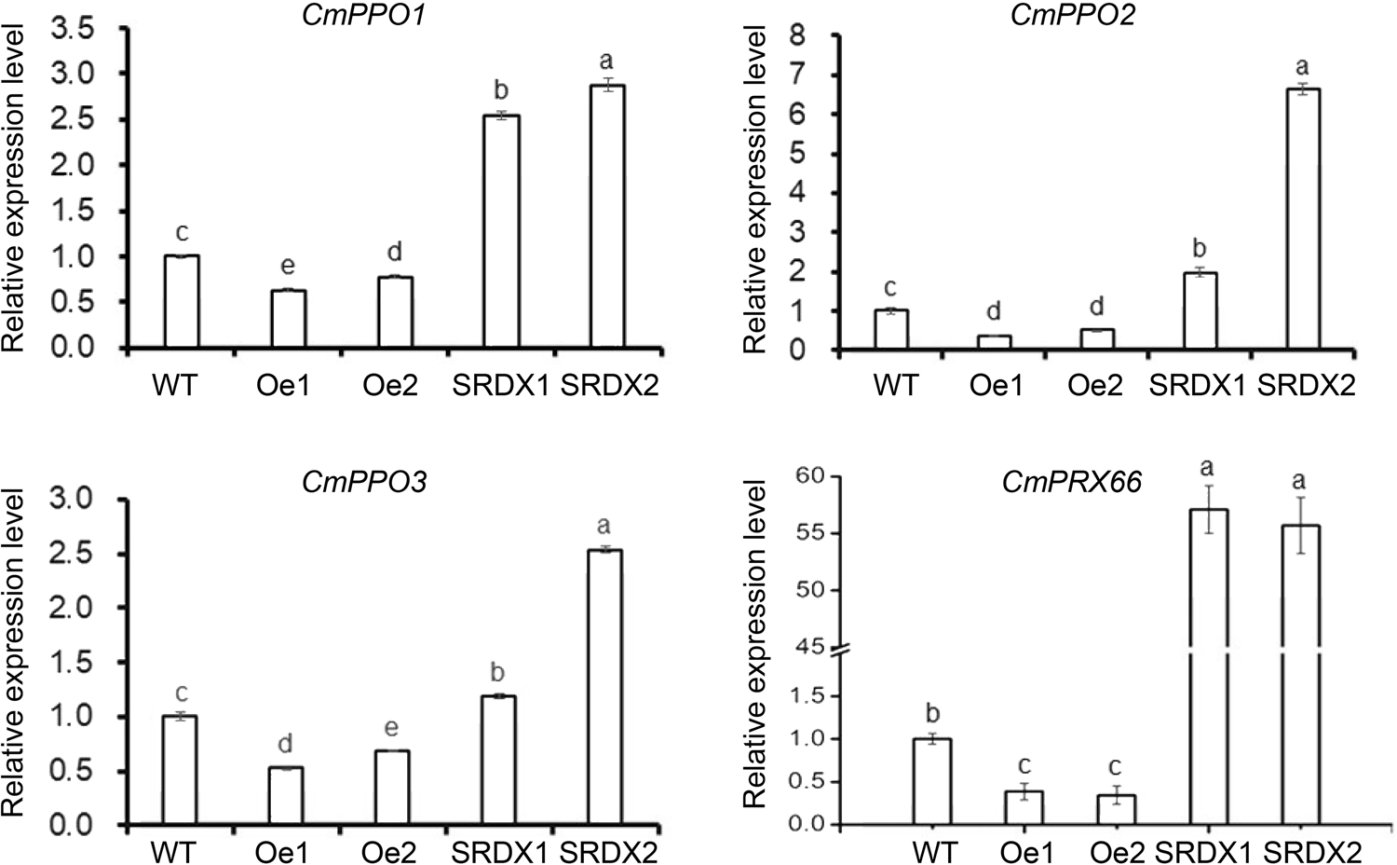
Differentially expressed genes (DEGs) involved in the secondary metabolism signaling pathway between WT plants and transgenic plants

Transcriptome analysis showed that there are 675 differentially expressed genes (DEGs) between *CmWRKY53-*overexpressing lines and wild-type plants and 404 DEGs between SRDX lines and wild-type plants. Compared with the wild-type plants, the *CmWRKY53-*overexpressing lines and gene-silenced lines shared 183 DEGs (Supplementary Fig. S1a). DEG_GO enrichment analysis showed that the expression levels of metabolism-related genes decreased in the overexpression plants compared to the wild-type plants, while they increased in the SRDX plants (Supplementary Fig. S1b). KEGG pathway enrichment analysis suggested that the DEGs whose expression changed in the transgenic plants are mainly related to the biosynthesis of isoquinoline alkaloids and phenylpropanoids (Table 2). The transcript levels of three polyphenol oxidase genes (*CmPPO1*, *CmPPO2*, *CmPPO3*) involved in the biosynthesis of isoquinoline alkaloids increased in pSRDX*-CmWRKY53* plants but decreased in the overexpression plants compared with the wild-type plants (Table 2). KEGG pathway enrichment analysis demonstrated that the peroxidase 66 gene, which is involved in phenylpropanoid biosynthesis, increased in the SRDX line but decreased in *CmWRKY53*-overexpressing plants compared to WT plants (Table 2). The expression levels of the abovementioned DEGs were verified by qRT-PCR (Fig. 7).

**Table 2:** KEGG pathway analysis of genes involved in secondary metabolites in WT and transgenic plants

Gene_id	Oe	WT	SRDX	annotation
Isoquinoline alkaloid biosynthesis				
c59656_g1	43.81	68.805	102.805	Polyphenol oxidase1
c45889_g1	399.425	578.175	818.65	Polyphenol oxidase2
c43930_g1	460.7	657.445	920.76	Polyphenol oxidase3
Phenylpropanoid biosynthesis				
c47749_g1	0.325	0.46	1.84	Peroxidase 66

## Discussion

### Structural characteristics and transcriptional activation activity of *CmWRKY53*

WRKY transcription factors are mainly characterized by the presence of the WRKYGQK domain in the N-terminus and a zinc finger structure in the C-terminus^12^. Despite WRKYGQK being a highly conserved region in the structural domain of WRKYs, it has been found that there are also variations in this sequence. These variations mainly occur for R, G, Q, and K, where the change of Q to E and K is the most common; for example, there are 19 variations in the rice WRKY domain structure: there are 7 of WRKYGEKs, 5 WRKYGKKs, and 1 each of WRICGQK, WRMCGQK, WKKYGQK, WIKYGQK, WKRYGQK, WSKYEQK and WRKYSEK. This genetic variation in the WRKY family in rice may due to the process of evolution^40^. AtWRKY53 and WRKY53 in wheat have conserved WRKYGQK domains^41^; however, a conserved WRKY domain was not observed in the WRKY53 protein from *Artemisia annua* (Fig. 1). Similarly, a conserved WRKY domain was not observed in chrysanthemum CmWRKY53, suggesting that CmWRKY53 might be evolutionally different from WRKY proteins in other species.

Our transactivation assay showed that the whole protein segment of CmWRKY53 is transcriptionally active. An N-terminal truncation assay showed that a protein segment ranging from 229 to 271 aa has transcriptional activity. Furthermore, a C-terminal truncation assay showed that an intact fragment of approximately 168 aa is important for transactivation activity (Fig. 3).

### CmWRKY53 negatively regulates the resistance of chrysanthemum to aphids

Previous studies have shown that a few members of the *WRKY* family play important roles in aphid resistance. Silencing of *SlWRKY70* attenuated the resistance of tomato to potato aphids (*Macrosiphum euphorbiae*), and *SlWRKY70* was reported to be required for *Mi-1*-enhanced resistance to aphids^42^. In *Arabidopsis*, *AtWRKY22* increases susceptibility to green peach aphids (*Myzus persicae*) via the suppression of salicylic acid signaling^43^. However, in wheat, silencing *WRKY53* increased the susceptibility to aphids while decreasing the expression level of *PAL*, and *PAL*-silenced plants are also susceptible to aphids, which implies that these genes operate via the same defense mechanism^41^. In the present study, *CmWRKY53* contributed to the susceptibility of chrysanthemum to aphids, which suggests that *CmWRKY53* might regulate aphid resistance in a way different from that of *WRKY53* in wheat.

### CmWRKY53-altered aphid resistance is potentially related to secondary metabolism

*WRKY*s affect a number of secondary metabolites, including phenylpropanoids, alkaloids, and terpenes, by regulating genes involved in metabolite biosynthesis^43–46^. *Brassica napus WRKY41-1* regulates the production of anthocyanin, and *WRKY23* regulates the biosynthesis of flavonols in *Arabidopsis*^44,47^. In potato, *StWRKY8* regulates resistance to late blight by regulating the isoquinoline alkaloid pathway^48^. *WsWRKY1* positively regulates the biosynthesis of phytosterol and triterpenoid withanolide accumulation and defense against biotic stress in *Withania somnifera*^49^. Here, transcriptome analysis showed that the identified differentially expressed genes between wild-type plants and *CmWRKY53* transgenic plants are mainly involved in secondary metabolites (Supplementary Fig. S1). Plants are able to overproduce reactive oxygen species (ROS) when they are challenged with insect feeding, and POD and polyphenol oxidase (PPO), which are involved in plant defense against insects and pathogens^50,51^. Increased activities of peroxidase and polyphenol oxidase enhance the resistance of cassava to the spider mite *Tetranychus urticae*^52^. Our previous study showed that peroxidase and polyphenol oxidase activity were enhanced by aphids, and the activities of polyphenol oxidase enzymes were increased in the resistant cultivars of chrysanthemum after aphid inoculation, suggesting that polyphenol oxidase might contribute to aphid resistance in chrysanthemum^53^. Transcripts of the PPO family encode PPO enzymes, which are characterized by a common central domain of tyrosinase, that can catalyze the hydroxylation of tyrosine, thereby forming DOPA, which is thought to contribute to the biosynthesis of benzylisoquinoline alkaloids^54,55^. PPO also contributes to lignification and produces polyphenols that reduce infestation rates of green peach aphids^56,57^. *PRX66* encodes a peroxidase involved in the lignification of tracheary elements in *Arabidopsis thaliana*^58^. In the present study, we found that genes encoding peroxidase and polyphenol oxidase decreased in *CmWRKY53*-overexpressing plants but increased in SRDX lines. Thus, we propose that *CmWRKY53* mediates sensitivity to aphids and that the mechanism could be related to plant secondary metabolism. However, more data need to be obtained before a definitive conclusion can be made.

## Conclusions

In conclusion, *CmWRKY53* was cloned from chrysanthemum, and its expression was induced by aphid infestation. The results showed that *CmWRKY53* mediates chrysanthemum susceptibility to aphids. The expression levels of secondary metabolite biosynthesis genes, such as peroxidase- and polyphenol oxidase-encoding genes, decreased in *CmWRKY53*-overexpressing plants, while they dramatically increased in *CmWRKY53*-SRDX plants. This suggests that the decreased levels of secondary metabolites in *CmWRKY53* contributed to the susceptibility of chrysanthemum to aphids.

## Supplementary information

Supplementary materials
