# Supplementary material for: Chrysanthemum CmWRKY53 negatively regulates the resistance of chrysanthemum to the aphid Macrosiphoniella sanborni
Source: Hortic Res. 2020 Jul 1;7:109. doi: 10.1038/s41438-020-0334-0 (PMC7327015; doi:10.1038/s41438-020-0334-0)
Supplement: Supplementary file 1 — Supplementary materials [file 41438_2020_334_MOESM1_ESM.docx]

**Supplementary materials**


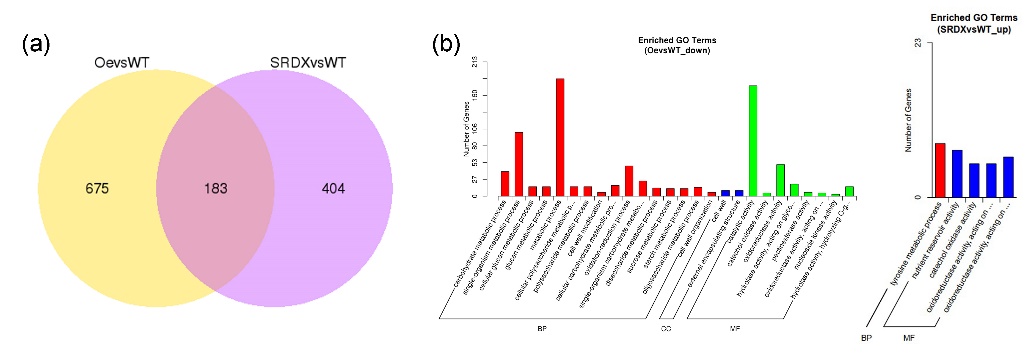


**Fig. S1** The RNA-seq analysis of WT and *CmWRKY53* transgenic lines (a): Venn diagram of DEGs of the comparison between WT and transgenic plants; (b): Gene Ontology (GO) functional classification of differentially expressed genes (DEGs).

**Table S1** Primer sequences used in the paper

| primer | sequence |
| --- | --- |
| CmWRKY53-ORF-F | AAGTCATCAGTGCATAATGGAGAGT |
| CmWRKY53-ORF-R | CCTCATTTATCTACGACTAATCATC |
| PPO1-RT-F | CGAGTCATCTCCCACCAAAAG |
| PPO1-RT-R | CCATGCCAGCCGTGTATTT |
| PPO2-RT-F | CCCAATAATGTAGACCGAAGGAA |
| PPO2-RT-R | TACAGCACCCTCCGACGTAAA |
| PPO3-RT-F | GAAGAGGCCGGCTGTGAAC |
| PPO3-RT-R | GAAAAACCACCCGCGTACTC |
| PRX66-RT-F | GGCATAGTGCAGGCCAGTTT |
| PRX66-RT-R | AGAATACGAGTCGGCAGGCTAA |
| 35S-F | GACGCACAATCCCACTATCC |
| SRDX-R | AGCGAAACCCAAACGGAGTTCTAG |
| EF1A-F | TTTTGGTATCTGGTCCTGGAG |
| EF1A-R | CCATTCAAGCGACAGACTCA |

**Fig. S1** The RNA-seq analysis of WT and *CmWRKY53* transgenic lines

**Table S1** Primer sequences used in the paper
